# Facilitative and obstructive factors in the clinical learning environment: Experiences of pupil enrolled nurses

**DOI:** 10.4102/curationis.v38i1.1263

**Published:** 2015-03-31

**Authors:** Eucebious Lekalakala-Mokgele, Ernestine M. Caka

**Affiliations:** 1 School of Health Care Sciences, University of Limpopo, Medunsa Sefako Makgatho Health Sciences University, South Africa; 2 The South African Military Nursing College, Military Hospital, Pellisier, South Africa

## Abstract

**Background:**

The clinical learning environment is a complex social entity that influences student learning outcomes in the clinical setting. Students can experience the clinical learning environment as being both facilitative and obstructive to their learning. The clinical environment may be a source of stress, creating feelings of fear and anxiety which in turn affect the students' responses to learning. Equally, the environment can enhance learning if experienced positively.

**Objectives:**

This study described pupil enrolled nurses' experiences of facilitative and obstructive factors in military and public health clinical learning settings.

**Method:**

Using a qualitative, contextual, exploratory descriptive design, three focus group interviews were conducted until data saturation was reached amongst pupil enrolled nurses in a military School of Nursing.

**Results:**

Data analysed provided evidence that acceptance by clinical staff and affordance of self-directed learning facilitated learning. Students felt safe to practise when they were supported by the clinical staff. They felt a sense of belonging when the staff showed an interest in and welcomed them. Learning was obstructed when students were met with condescending comments. Wearing of a military uniform in the public hospital and horizontal violence obstructed learning in the clinical learning environment.

**Conclusion:**

Students cannot have effective clinical preparation if the environment is not conducive to and supportive of clinical learning, The study shows that military nursing students experience unique challenges as they are trained in two professions that are hierarchical in nature. The students experienced both facilitating and obstructing factors to their learning during their clinical practice. Clinical staff should be made aware of factors which can impact on students’ learning. Policies need to be developed for supporting students in the clinical learning environment.

## Introduction

Military health nurse training differs from general nurse training in South Africa. Nurses are usually trained in private or public schools and colleges of nursing for a period of two years (for enrolled nurses) and four years (for comprehensive basic nursing) (South African Nursing Council 1975; 1993a; 1993b). The military in South Africa train their own enrolled nurses within the military schools of nursing for two years as pupil enrolled nurses (PEN), although under the same South African Nursing Council regulation 2175 for the training of regular enrolled nurses (South African Nursing Council 1993a). The military PEN commence with a six-month basic military training to prepare them as soldiers first before they embark on a two-year nursing programme, on completion of which they are registered as enrolled nurses.

Maginnis and Croxon (2007:218) insist that the clinical placement of students needs to prepare them adequately for practice in an environment that is constantly changing. Whilst nurses are generally trained in corresponding private and public clinical learning environments (CLEs), military nurses receive their own clinical learning experiences in the military health and public health institutions, for diverse clinical placement and to be exposed to different health care experiences that will equip them to nurse holistically (Purdie, Sheward & Gifford 2008:315).

This arrangement of placing military students in public health institutions was made because military hospitals were initially used only as a sickbay (a room for treating sick and injured soldiers) and for visiting the doctor on an outpatient basis. They therefore lacked diverse clinical exposure for their students. These students, including PEN, have to experience different challenges in two diverse CLEs. In the military health system only members of the military (various ranks) and their family members may be admitted as patients, whereas the public health sector accommodates civilian patients from all of the different spheres of South African society (Caka & Lekalakala-Mokgele 2013:2).

### Problem statement

The CLE constitutes a clinical setting which offers the students an opportunity to practise the necessary skills to enable them provide patient care (Cheraghi *et al*. 2008:26). The uniqueness of the CLE in the military health service means that the experiences of the PENs in the military are different from those in the public sector. Firstly, their workplace learning takes place in a military milieu where power ranks are more pronounced. It is where they may have to treat high-ranking personnel as their patients and have to also contend with high-ranking clinical staff who are often also trained in military nursing and military culture. Secondly, because of the limited number of clinical areas in the military health service, students are allocated to public hospitals where they take care of civilian patients under different conditions than exist in the military setting.

Literature reveals that there are both facilitative and obstructive factors to students’ learning within this environment (Löfmark & Wikblad 2001:49). The researcher's personal experience of five years as an educator in the military health service, as well as her concern about the need to understand the dynamics that facilitate or obstruct learning, led to initiation of this project in order to investigate the experiences of PEN in the military health service. This has not been done previously in South Africa and it was not known how these PEN would describe their experiences in these two diverse clinical settings. The following research question was asked: ‘How do military PEN experience their clinical learning environment?’

### Definition of key concepts

#### Clinical learning environment

In this study, the CLE is a learning environment accredited by the South African Nursing Council where students acquire the knowledge and skills to manage both military patients of various ranks and their family members. It also includes the public health sector which accommodate civilian patients from all spheres of South African society.

#### Military health setting

This is a formal setting for the development of various skills, including clinical skills, that will prepare PEN in times of war and defence of the country. PEN are trained to care for sick or injured soldiers and their families in the line of battle.

#### Public health setting

A setting that mobilises local, State, national and international resources to ensure optimal conditions for people's health. It comprises preventive, curative and rehabilitative services (Basu, Jina & Naidoo 2008:7).

#### Pupil enrolled nurses

This term refers to persons undergoing a two-year programme at an approved nursing school, who have complied with the prescribed conditions and have furnished the prescribed particulars as required by section 24 *x* of the *Nursing Act* 50 of 1978 (Republic of South Africa 1978).

### Literature review

Literature suggests that nursing students’ clinical placement experiences are often fraught with longitudinal and multidimensional challenges (Clare *et al*. 2003). These challenges include a lack of support of students (from both the CLE staff and their facilitators), which can lead to missed opportunities in terms of teachable moments (Newton, Billet & Ockerby 2009). Reports suggest that nurses deal more with documentation than patient care – the feeling that there is a lack of interpersonal relationships between students, staff members and the patients is also documented (Pearcey & Draper 2008:595). The CLE is even recognised as a source of stress that creates feelings of fear and anxiety, which in turn affect the students’ responses to learning (Chesser-Smyth 2005:320).

According to Levett-Jones and Lathlean (2009:348) students can experience the CLEs as belligerent if they are constantly criticised and judged harshly by clinical staff. For many students, clinical placements are typified by a feeling of alienation and a lack of belonging (Goh & Watt 2003:14; Newton *et al*. 2009:633). These behaviours can be obstructive to learning. According to Löfmark and Wikblad (2001:46), the CLE is obstructive to learning when there is no student–supervisor relationship and/or when supervisors make condescending comments, are irritated or not interested in students and do not allow students to take part in patient care activities.

Violence is another factor that is obstructive to learning. Carley and Mackway-Jones (2005:26) suggest that nursing profession is hierarchical and is embedded in the military system. This hierarchy results in student nurses experiencing ‘horizontal violence’ (Curtis, Bowen & Reid 2007:156), with junior members in the profession being bullied, ignored and disrespected.

Facilitative factors are considered to be those that promote learning. Literature has identified multiple such factors, amongst others a sense of belonging and acceptance. A number of affective consequences are said to influence the student's sense of belonging, including feeling safe, comfortable, satisfied and happy (Levett-Jones & Lathlean 2008:103). A sense of belonging is underpinned by a need for affirmation, friendship and being part of the group (Friedkin 2004:410). When students realise that they are not part of the collective, they may present with low self-esteem and unhappiness, which may result in a diminished learning process (Baumeister, Twenge & Nuss 2002:817). It is thus important to inculcate a sense of belonging that produces happy group members, because students who feel ostracised by their peers or those in authority will not function optimally (Tabane & Human-Vogel 2010:504).

According to Löfmark and Wikblad (2001:47), being allowed to take responsibility and to work independently, having opportunities to practise tasks and receive feedback, collaborating with staff and supervising others, gaining an overview of the setting and a sense of control, are all factors considered to facilitate learning in the CLE. Levett-Jones *et al*. (2007:211) assert that a feeling of acceptance, being valued and being involved with others are some of the attributes of belonging. The purpose of this article is to explore and describe PEN experiences of facilitative and obstructive factors in clinical learning in two diverse settings.

## Research methods and design

### Design

An explorative, descriptive, contextual qualitative research approach was used in this study to explore and describe the experiences of PEN in the military health CLE and within public health settings.

### Population and sample

There was a total of 30 PEN in the studied institution at the time of collecting data for this study, which included PEN in their second year of training. The second-year PEN have been in training for over a year and have been more exposed to the various clinical learning situations, including placements in public hospitals. Criteria for inclusion in this study were that participants should be registered as PEN in the Military Nursing College, must be in their second year of study and should have been allocated to the military health CLE and public hospitals. The final sample size, using the eligibility criteria, was determined by data saturation (Streubert & Carpenter 2011:90).

### Data collection method

Data were collected by means of focus group interviews. A total of 19 students participated in the study voluntarily. Three focus group sessions were conducted, with two groups consisting of six members and a third group consisting of seven members. An expert in the facilitation of focus groups was approached to act as a moderator for the sessions. Appointment of the moderator was important, as the researcher was a facilitator in the nursing college and known to the students, so this allowed participants to participate freely in the study. The moderator was a specialist psychiatric nurse skilled and experienced in focus group management. She is also an expert in qualitative research. The researcher made arrangements for the venue (a classroom in the Military Nursing College) in which to conduct interviews. The researcher also clarified the process of focus group interviews to the participants, as well as the expectations of the moderator during the interviews and how the interviews would be conducted. Data were recorded using a tape recorder with the permission of participants and field notes were handwritten by the researcher to capture actual discussions (Polit & Beck 2005:307). Participants’ reactions and attitudes were also observed and noted throughout the interviews. Data were collected until saturation was reached (Streubert & Carpenter 2011:90–91).

### Data analysis

The data were analysed according to Henning's four-step method (2004:126). Tape-recorded interviews were listened to as soon as the interviews had been completed and these were then transcribed verbatim. The researcher recorded and organised the data on note cards and audiotapes were labelled to ease data retrieval. Cross-checking of data with each note card was performed to keep track of each piece of data collected. Units were then organised into a system derived from the data. After the data were organised they were ready to be categorised into themes and sub-themes. The researcher and co-coder created codes from the data independently; extracted codes were then compared for similarities and differences. Where differences occurred these were resolved, leading to emergence of the themes and sub-themes presented in the findings. The co-coder was a specialist in analysing and coding qualitative studies.

### Context of the study

The study was conducted in a Military Nursing College, of which there are three in South Africa. This college is situated in an urban area in one of the nine provinces in South Africa. A quiet classroom in one particular college was organised by the researcher in which to collect data without distraction.

## Ethical considerations

Approval to conduct this study was obtained from the Ethics Committee of the Faculty of Health Sciences of North-West University (Potchefstroom Campus), the Military Ethics Committee and the Officer Commanding of 3 Military Hospital. A written letter of request was given to the students, inviting them to participate in the study. Participants were informed in full about the study and were asked to provide written consent to participate. They were informed that the research did not form part of their work and that they could withdraw from participation at any time during the process without being disadvantaged in any way. All participants were assured that confidentiality would be maintained as only the researcher had access to the audiotaped material. Audiotapes were kept locked in a safe place and erased after the research was completed. The use of real names was avoided.

### Trustworthiness

Trustworthiness was maintained by using strategies of credibility, transferability, dependability and confirmability (Lincoln & Guba 1985:290). The research methodology and research context were described in detail to enable interested researchers to transfer these to other studies. The results and findings of the research process, including raw data, field notes, data reduction and analysis products, and theoretical notes relating to trustworthiness were kept as an audit trail of what transpired during the research process to ensure confirmability. Dependability was achieved through clear description of the exact methods of data collection, analysis and interpretation. The appointment of an independent coder with field research expertise also assisted in ensuring dependability. Prolonged engagement with the data assisted the researcher in identifying recurring patterns and themes, thus ensuring credibility.

## Results

This study set out to describe PEN experiences of facilitative and obstructive factors in clinical learning in two diverse settings. Two main themes emerged from the analysis: factors facilitating learning and those obstructing learning. Self-directed learning and acceptance by staff were experienced as facilitating PEN learning, whilst non-acceptance by staff, wearing of military uniform in public hospitals and workplace violence were experienced as obstructive to learning.

### Factors facilitating learning

#### Self-directed learning

The limited number of learning opportunities in the military health setting afforded students ample time to practise procedures on these few available opportunities and to be in control of their own learning. The ability to control their own learning was perceived as empowering: they could make decisions and enjoy learning as they did not feel pressured to complete tasks but could work at their own pace. This practice made students feel that they are trusted to make judgements about patient care, which facilitated learning. This was represented in the following excerpts:

‘Being a military nurse works at advantage because we are only serving military members and their family, so we don't have too much pressure on us, we learn at our own pace and given time to learn all of those stuff.’ (PEN 1)

‘They give us the opportunity to find solutions for patients’ problems on our own and to make good judgements.’ (PEN 8)

‘Here at the military … we are given topics to prepare and present, we look for information ourselves; in that way we get to know what is happening in the ward.’ (PEN 14)

#### Acceptance by staff

Acceptance is another facilitating factor experienced by the students in this study. They expressed that they felt accepted by the doctors and nurses in the public CLE as they received good guidance and supervision from them in the wards of the public hospitals. This created confidence and competency in implementing nursing procedures, as the supervisor was always around. The following excerpts validate this:

‘In the public hospital they accept us as part of the team, and the doctors will explain everything about the patient. We will do everything under supervision of the doctor.’ (PEN 2)

‘In the provincial hospital the sister calls you and teaches you the procedure.’ (PEN 7)

‘When we are in the outside hospitals and there are procedures that they want us to learn more about, they will call us to watch.’ (PEN 10)

### Factors obstructive to learning

#### Non-acceptance by staff

One respondent said that they did not always feel accepted, and was made to feel that they were always in the way, particularly in the military CLE:

‘Sometimes they will become angry, saying students are just causing traffic. How could I learn when they don't accept us?’ (PEN 11)

#### Wearing military uniform in public health settings

Wearing military uniform when allocated to public hospitals was perceived as being obstructive to learning. PEN explained that they felt marginalised when referred to as soldiers and not nurses by some staff members. Their perception was that they were easily blamed for everything that went wrong because they wore a military uniform. The participants also alleged that some staff members often made condescending remarks about them and caused them a lot of embarrassment. Patient discomfort was also cited. Their views were stated as follows:

‘Sometimes in public hospitals we feel bad as we wear military uniform, because they will call us soldiers and sometimes securities. They are sometimes not nice.’ (PEN 9)

‘Maybe something was not done, they will say “it's the soldiers”; it is always a pain for us, they call us soldiers and not nurses. So it's not nice as this embarrasses us.’ (PEN 2)

‘Patients are also not comfortable when we have to do procedures on them. They do not see us as nurses, they call us soldiers because of our uniform.’ (PEN 15)

#### Workplace violence

Participants expressed that they were often subjected to different forms of violence. These were mostly non-physical in nature, in the form of shouting, bad mouthing, being blamed and felt punished if one person was in the wrong. Some felt that military rules are applied in nursing and perceived this in a negative light:

‘I just want to add on that issue of the shouting, wherever you go as long as you in the department they will shout.’ (PEN 5)

‘They call us, but when they call the college, stating how bad we are. The only thing we will hear from them, is how lazy we are, behind the staff nurses’ back, they tell us we work more than them. They talk about us to the staff nurses, when those staff nurse are not there they talk about them.’ (PEN 16)

‘One of the old nurses forgot to take out the temperature, I mean, the thermometer, then when we were supposed to change the patient, eh, to give the patient a bed pan that thermometer fell and it broke, the glass was all over the bed. They blamed the students, even though we came after 3 [*pm*], they blame the students. If anything goes wrong they blame the students.’ (PEN 13)

‘He started swearing at me, verbally abusing me. I then called the police. I opened a case of assault.’ (PEN 14)

‘The military rules are applied in nursing and it is very destructive. When one student makes a mistake, everybody is punished.’ (PEN 6)

One of the participants in this study was physically assaulted by a high-ranking member whilst carrying out his nursing duties:

‘A rank is misused in the military; I was physically assaulted by a high-ranking member whilst carrying out my nursing duties.’ (PEN 19)

## Discussion

Findings show that the students experienced the CLE as both facilitating and obstructive to their learning. Compared to the military setting, students experienced the public CLE to be more facilitative as they felt empowered, could self-direct their clinical learning and had support from the clinical staff. Silén and Uhlin (2008:462) are of the opinion that ‘students’ feelings of being in charge and having a genuine impact on the learning situations are crucial for their desire to take responsibility’. Compton (2005:36) asserts that there is a strong linkage between self-directed learning and positive psychology, and that attributes such as perceived control and satisfaction are important for self-directed learning to occur. Turner (2007) concurs that ‘self-directed learners demonstrate greater awareness of their responsibility in making learning meaningful and monitoring themselves’, thus making them more effective learners and social beings. According to Löfmark and Wikblad (2001:49), these positive attributes are said to be facilitating factors for learning in the clinical setting; these authors showed in their study that students value independence as a facilitating factor.

The second finding was that some participants experienced acceptance in the public setting where the staff was supportive and willing to supervise them. Several authors posit that it is important that students fit in and are accepted as part of the group during clinical placement, as this kind of welcoming environment is a source of active participation and provides learning opportunities (Hartigan-Rogers *et al*. 2007:1; Levett-Jones & Lathlean 2009:348; Nash, Lemcke & Sacre 2009:48). Staff members need to be available, friendly and willing to teach in order to construct a climate conducive to learning (Croxon & Maginnis 2009:240).

In their study, Levett-Jones and Lathlean (2008:107) found that ‘students felt more empowered and enabled to capitalise on the available learning opportunities when they felt they had a legitimate place in the nursing team’; conversely, if not accepted, they can find it difficult to learn. Individuals’ perceptions about their participation and level of acceptance in the group influence their sense of belonging (Tabane & Human-Vogel 2010:493). This sense of acceptance must be fostered, as learners who feel ostracised by those in authority may present with low self-esteem and unhappiness. If the PEN (learners) feel that they are not part of the collective, their functioning may not be optimal, resulting in a diminished learning process (Baumeister *et al*. 2002; Tabane & Human-Vogel 2010:504). According to Chesser-Smyth (2005:320), the morale of students can be boosted if they are welcomed, respected and valued and, as a result, they become more eager to learn.

It is also evident that students are not always accepted in the military CLE. Such behaviour is identified as being obstructive to learning (Hathorn, Machtmes & Tillman 2009:229).

Studies have shown that a lack of support in the CLE can be a source of anxiety and stress for students (Elliott 2002:72; Levett-Jones & Lathlean 2008:109; Papastavrou *et al*. 2009:176; Shipton 2002:244). Literature cites different reasons for a lack of support for students in the clinical setting, including registered nurses seeing facilitation of learning or supervision as extra work for them and the unavailability of staff (Clarke, Gibb & Ramprogus 2003:106; Mongwe 2001:4).

The wearing of military uniform was perceived as obstructive to learning by participants in the study. They claimed that the uniform caused them embarrassment, that they often felt marginalised and were blamed for multiple wrongs regarding patient care. Studies have shown that CLEs can be very traumatic for students when they are exposed to harsh remarks and made to feel like outsiders (Hathorn *et al*. 2009:242; Levett-Jones & Lathlean 2008:105; Rush, McCracken & Talley 2009:315).

The hierarchy within both CLEs made the PEN experience of clinical learning more challenging and was perceived as being obstructive to learning.

This study shows that participants were exposed to some form of workplace violence in the CLE. Workplace violence has many names in nursing literature. According to Mendez (2011:2), the phenomenon is known as horizontal violence, lateral violence, bullying, and intrapersonal workplace aggression. I the context of nursing, lateral violence has been defined as ‘nurse-to-nurse aggression’ (Griffin 2004:258). According to the literature, lateral violence finds expression in physical assault, psychological abuse and non-physical violence, such as verbal abuse, mobbing, gossip, jealousy, blaming, yelling, insulting, name-calling, lack of respect, bullying and intimidation, backstabbing, unfair criticism and defamatory statements about individuals or groups, whether it comes from nurses or other healthcare professionals (Bartholomew 2006:4–5; Brooks & Phillips 2013:9; Edwards & O’Connell 2007:27; Felblinger 2009; Gimeno *et al*. 2012: 30; Nachreiner *et al*. 2007:674)

Horizontal violence is defined as ‘[*r*]epeated, offensive, abusive, intimidating, or insulting behavior, abuse of power, or unfair sanctions that makes recipients upset and feel humiliated, vulnerable, or threatened, creating stress and undermining their self-confidence’ (Vessey, DeMarco & Budin, as quoted in Sellers *et al*. 2009–2010:21). According to King-Jones (2011:80), horizontal violence includes forms of non-physical intergroup conflict that manifest in overt and covert behaviours of hostility ranging from intimidating body language to sarcastic comments, fault finding, belittling gestures, discourtesy, disinterest and discouragement, amongst other behaviours.

PEN were also subjected to physical violence. One of the participants was physically assaulted by a high-ranking member who was not satisfied with the treatment he received. According to Thomas and Burk (2009:229), this conduct can affect the working environment of the demoralised students and impede their learning. Experiences that have been described by PEN are related to the two very different roles – being a soldier and being a nurse – which causes the students to experience conflict. Fundamentally, one role is about force, aggression and violence whereas the other is about caring for the sick and the injured. It is not established if they have been prepared for these dual role.

## Limitations

There are three military nursing colleges in South Africa and the findings of the study are limited to the opinions of only one group of second-year students at one of these. It is therefore important to conduct future studies on students in other colleges.

## Conclusion

This study indicates that students experience both facilitating and obstructing factors to their learning during their clinical practice. The findings indicate that learning in the clinical setting is facilitated when students take responsibility and control of their learning and their self-confidence increases. Students feel safe to practise when they are supported by the clinical staff. They feel that they belong when the staff shows interest in them and welcomes them.

The study also showed that learning is obstructed when students are not accepted and are met with condescending comments or are blamed and embarrassed by the clinical staff. The lack of supervision in the military setting further obstructed their learning. Wearing of military uniform during the public clinical learning seemed to be problematic for the PEN. It was also evident that students experienced some forms of violence in the CLEs. Violence in any form is not conducive to learning and will be obstructive. Students cannot effectively be prepared clinically if the environment is not conducive to and supportive of clinical learning. Whilst research in this setting brought the experiences of military PEN to the fore, similar studies on non-military PEN may be useful in order to compare similarities and differences, with a view to using such information to improve the teaching and learning environment of students.

### Recommendations

Based on the conclusions of the study, it is recommended that students be allowed to take responsibility and initiative for their learning, as this can promote learning. It is also recommended that PEN should not be compelled to wear military uniform in public hospitals and should be allowed to dress in similar uniforms to their counterparts, so that they are not treated differently when allocated to the CLE of public hospitals. Clinical staff should be made aware of the impact that condescending remarks may have on students’ learning. They should also recognise the need to support actively and welcome students so as to enhance the working environment. Policies on managing violence in the clinical area need to be developed, with specific attention on how this may affect students, particularly when they are dealing with high-ranked officers. There is also a need to prepare students for the dual role of being a nurse and a soldier so that they are able to experience the CLEs in a positive light.
